# PB.30. Apparent diffusion coefficient and fractional anisotropy values as biomarkers for treatment response in breast cancer

**DOI:** 10.1186/bcr3730

**Published:** 2014-11-03

**Authors:** EA Joyce, AJ Fagan, JP McMorrow, D Byrne, MJ Kennedy, JF Meaney, SA O'Keeffe

**Affiliations:** 1 Centre for Advanced Medical Imaging, St James's Hospital, Dublin, Ireland

## Introduction

The aim was to evaluate whether changes in apparent diffusion coefficient (ADC) and fractional anisotropy (FA) values predict early response in patients receiving neoadjuvant chemotherapy (NACT) for breast cancer and to assess the effect of tumour marker clip placement on ADC values.

## Methods

Twenty patients with invasive breast carcinoma underwent MRI at three time points: at baseline (TP0) and following the first (TP1) and second (TP2) cycles of NACT. 3T MRI (Achieva; Philips) was performed using a standard protocol including diffusion-weighted and diffusion tensor imaging. Baseline and sequential data in responder and nonresponder groups were compared. To assess the effects of a commercially available titanium-hydrogel clip on ADC values, DWI was performed on a phantom consisting of a clip embedded in a tumour-mimicking target.

## Results

At baseline, mean tumour ADC (0.92 × 10^-3 ^mm^2^/second) was statistically lower than disease-free fibroglandular breast tissue (1.75 × 10^-3 ^mm^2^/second) (*P *< 0.0001). Mean FA values of tumour (FA = 0.139) and disease-free tissue (FA = 0.135) were similar. Compared with baseline values, tumour ADC of responders significantly increased at TP1 (*P *< 0.0001) and TP2 (*P *< 0.0001) while a significant increase in tumour FA of responders was seen at TP2 (*P *< 0.008). No statistical change occurred in tumour ADC or FA values of the nonresponder group. The ADC value of the clip in the phantom (1.7 × 10^-3 ^mm^2^/second) was higher than the ADC value of surrounding tumour (1.4 × 10^-3 ^mm^2^/second).

## Conclusion

Changes in ADC and FA values early in the course of treatment may predict response in patients receiving NACT for breast cancer. The gel-containing clip used for tumour marking prior to NACT results in a source of error when calculating tumour ADC values and should be avoided when drawing regions of interest.

